# Financial Performance Under the Influence of the Coronavirus Disease 2019: Effects of Strategic Flexibility and Environmental Dynamics in Big Data Capability

**DOI:** 10.3389/fpsyg.2021.798115

**Published:** 2021-12-20

**Authors:** Limei Chen, Liping Zhai, Weiwei Zhu, Gongzhi Luo, Jing Zhang, Yaozhen Zhang

**Affiliations:** School of Management, Nanjing University of Posts and Telecommunications, Nanjing, China

**Keywords:** COVID-19 pandemic, big data capability, strategic flexibility, financial performance, environmental psychological dynamics

## Abstract

This study draws on the dynamic capabilities view and the firm’s big data capability (BDC) in the new economic environment. It constructs an adjusted intermediary model to study the mechanism of BDC, strategic flexibility, and environmental dynamic affecting financial performance. We find that strategic flexibility plays an intermediary role in the “Converse-U” relationship between BDC and financial performance. Environmental dynamics adjust the relationship between BDC and financial performance positively and smooth the “Converse-U” relationship. The findings suggest building and managing BDC, combining BDC with the management process, and achieving continuous financial performance improvement in a dynamic environment. The paper also puts forward the nonlinear hypothesis, discusses the “Converse-U” relationship between BDC and enterprise financial performance in the Chinese context of digital economy explosion and growth, and considers the intermediary mechanism of strategic flexibility and the regulatory effect of environmental dynamics.

## Introduction

The large-scale outbreak of coronavirus disease 2019 (COVID-19) in early 2020 posed a huge challenge to the survival and development of enterprises. In the first quarter of that year, GDP decreased by 6.8% year-on-year, and industry, investment, and consumption indicators dropped significantly. To eliminate the dilemma of delayed resumption of work and plummeting income, most enterprises have to speed up digital transformation and rely on Internet technology and big data application to upgrade the business structure. Technological progress has promoted online digitisation and intelligence, and enterprises have carried out informatisation reform and industrialisation transformation with the assistance of cloud computing and big data platforms. In the new context of the explosive growth of the digital economy, the realisation of the enterprises’ financial performance depends on enterprises that can successfully implement the digital transformation and big data capability (BDC) building, so the BDC directly affects the achieving form of enterprises financial performance (Patrick and Adamantia, 2017). As one of the dynamic capabilities for businesses to grow and gain sustainable competitive advantages, what is the impact of BDC on financial performance? The research on this issue has increasingly been brought into focus by academic and business circles (Yu, 2018). Although some relevant researches on the relationship between BDC and financial performance have been constructed, there are still some puzzles to be solved.

On the one hand, it has been suggested that enterprises with BDC can quickly iterate on products and services, achieve personalised marketing methods, and complete pricing systems, thus contributing to the enhancement of enterprises’ the financial performance (Akter and Wamba, 2016; Gupta and George, 2016; Yaqoob et al., 2016; Côrte-Real et al., 2017; Mikalef et al., 2019). Because the enterprise’s management ability and digital technology fail to realise coordinated development, the digital technology cannot integrate with the original resources and business, thus inhibiting the improvement of enterprise financial performance (Vidgen et al., 2017; Guoqing et al., 2018). Two main reasons for the inconsistency of research conclusions and the ambiguity of explanation are as follows: First, there may not be a simple linear relationship between BDC and financial performance. Previous studies were based on the fact of a linear relationship between the two, and this assumption may be the reason for the inconsistency of research conclusions; Second, previous research which are mainly focused on the direct impact of BDC on financial performance, possibly ignoring the different mechanisms and contextual factors, exist in the relationship between BDC and financial performance, so it is urgent to explore the intermediary variables and adjustment mechanisms between the two.

According to dynamic capability theory, as one of the significant dynamic capabilities in the digital economy, BDC has a lasting and profound impact on enterprise strategy realisation and financial performance advance (Teece, 2017). Especially against the backdrop of COVID-19, BDC is playing an increasingly significant role in enhancing the resilience of supply chains, improving the level of digital application, and resolving the operational and financial crisis. BDC is considered a vital factor affecting strategic flexibility (Han and Zhang, 2021). Strategic flexibility can enhance the ability to cope with environmental changes and maintain high agility and flexible allocation of resources so that enterprises can quickly respond to varieties and then coordinate the elements when facing the risks arising from environmental changes, thus maintaining a long-term competitive advantage. Strategic flexibility has become an indispensable condition for an enterprise’s success (Correa et al., 1994; Kaufman, 2015; Raguseo and Vitari, 2018). Thus, great importance is attached to strategic flexibility by managers. However, in the context of China, the mechanism of BDC on strategic flexibility has not been dived into, and research on the relationship between BDC and financial performance from the perspective of strategic flexibility is even more absent. The core connotation of BDC includes the capability of enterprises to apply big data technology to integrate resources, realise internal process optimisation and external competitive trend prediction, and adapt to environmental changes. Enterprises with moderate BDC can filtrate, analyse, and mine a large number of complex and heterogeneous data through big data technology to obtain data value and continuous competitive advantage.

In contrast, insufficient BDC, like deficient ability to integrate, analyse, and utilise big data resources, will make it difficult for enterprises to capture potential business opportunities, which is not conducive to forming strategic flexibility. Combining with the existing theories and researches, this paper holds that strategic flexibility is an important intermediary mechanism of BDC affecting financial performance. Furthermore, the process of the enterprise BDC acting on financial performance is affected by complex contextual factors. When the environmental dynamics are low, the enterprise’s market demand, technical changes, and institutional environment are stable and predictable. However, in the highly dynamic environment, just like the current epidemic environment, a series of domestic and foreign environmental uncertainties, including the decline and delay of consumer demand, is increasing. Thus, the risk of business interruption and operation crisis faced by the enterprise is also rising (Chen and Liu, 2020). Owing to the unpredictability of the market, technology, and institutional environment, more resources need to be reserved by enterprises to deal with external shocks (Duanmu et al., 2018). Enterprises with high BDC can better brace themselves for a period of upheaval based on in-depth analysis of internal and external data. By rapidly mobilising and allocating resources to adjust products and services ahead of competitors (Vela-Jiménez et al., 2014), enterprises can seize the market opportunity to quickly launch new products or services that cater for customers and, finally, conduce to the acquisition of a lasting competitive advantage, thereby improving the financial efficiency (Yunfeng et al., 2019). Therefore, environmental dynamics will be considered a scenario condition in studying the relationship between BDC and financial performance.

The contribution of this research to the current mainly includes the following three points: First of all, this paper puts forward the nonlinear hypothesis, discusses the “Converse-U” relationship between BDC and enterprise financial performance, which offers a possibility of researching the relationship between the two; In addition, this paper explores the mechanism of BDC affecting performance in the Chinese context of digital economy explosion and growth and considers the intermediary mechanism of strategic flexibility and the regulatory effect of environmental dynamics. Finally, the research emphasises the value of BDC to organisation management under the background of the post-epidemic era, and the research conclusion has a significant value for optimising enterprise BDC, constructing strategic flexibility and booting enterprise performance.

## Theoretical Background and Research Model

### Big Data Capability and Financial Performance

Financial performance refers to the cost control and profitability of an enterprise based on existing business operations. BDC can realise more accurate identification of customer needs, the faster segment of the target market, and product iterations through personalised marketing, accordingly showing the better financial performance (Akter et al., 2016). Dynamic capability theory suggests that enterprises should enhance the alignment with a dynamic environment to quickly respond to changes in the market and external technology (Adner and Helfat, 2003). The theory emphasises that enterprises can allocate and update existing resources according to needs in the dynamic environment and quickly deal with market and business environment changes. As one of the dynamic capabilities, BDC plays a crucial role in all aspects of enterprise management, inevitably affecting financial performance.

Current connotation understanding and dimension classification of BDC is very diversified. Wikipedia defines big data as a collection of data that cannot be captured and managed by regular software tools over time (Lynch, 2008). As the core capability in the digital economy, research on BDC is carried out in many fields. In the perspective of the technology field, BDC is considered to exist in big data acquisition, integration, analysis, and visualisation technology (Wang and Hajli, 2017). Through the collaboration of big data technology and relevant infrastructure, valuable decision-making and dynamic management capability can be obtained (Wang et al., 2017). From an organisational perspective, a system based on BDC is constructed and applied to management practice based on viewpoints on organisational structure, organisational culture, and organisational knowledge (Corte-Real et al., 2017). User heterogeneity is proposed based on user cognition (Orlikowski, 2007). It is pointed that human, technology, and organisation, together, make up the relational ontology and form dynamic BDC from environmental interaction perspective (Orlikowski and Scott, 2008; Sivarajah et al., 2016). Researchers in strategic management have developed the BDC scale (Cecez-Kecmanovic et al., 2014; Akter et al., 2016; Wamba et al., 2017), which lays a foundation for empirical research from the resource-based view and dynamic capability view (Ayabakan et al., 2017; Nan et al., 2018). In this paper, it is considered that BDC is the dynamic capability of enterprises to filtrate, integrate, reconstruct, and utilise massive internal and external data resources and transform them into unique advantages to support enterprises’ strategic transformation and operation decisions in the changing environment (Rijmenam et al., 2018). BDC is a distinct and pivotal resource in the digital economy and a dynamic capability for practice. This dynamic feature integrates internal and external resources to achieve original competitive advantages (Caifeng et al., 2019). Weihong et al. (2016) divide BDC into three dimensions: big data acquisition integration capability, big data in-depth analysis capability and big data insight, and prediction capability. Among them, big data acquisition integration capability emphasises the ability of enterprises to collect, integrate, and continuously update internal and external big data resources. Big data in-depth analysis capability requires enterprises to analyse and mine massive data and convert them into unique advantages. Big data insight and prediction capability refers to monitoring changes in the market environment and predicting future trends.

As the new functioning capital and vital resource of enterprises, the strategic significance of big data lies in the professional processing of massive data, providing necessary real-time information for enterprise decision-making, realising accurate prediction of customer and market demand, and maintaining the long-term competitive advantage of the enterprises. It is ultimately reflected in improving financial performance, including profit growth, business development, and advance in return on assets (Harris and Davenport, 2007; Barton and Court, 2012; Chen et al., 2015). Above all, big data resources include internal operation and external environmental data of the enterprise and non-data resources such as infrastructure, human resources, and technical resources. Enterprises utilise diversified data platforms and complete infrastructure to achieve resource integration, filter, store, process, and professional value from massive data with complex structures, and guide internal business and external market strategies, enhancing enterprise market value. Wamba et al. (2017) confirm that BDC directly affects financial performance and market performance. But excessive big data resources and integration often make enterprises pay more attention to emerging resources and ignore the adaptability of original resources.

Next, the analysis capability is to extract value from huge amounts of data and gain new insights. Surveys of executives find that the higher-performing organisations use five times more often than lower-performing organisations (Hitt et al., 1998). Big data analysis empowers enterprises to know about product operation and user reviews to optimise user experience, maintain customers, and analyse internal business processes and optimal directions based on large amounts of data. The more professional analysts and technologies are, the more sensitive they will be to discovering the potential value of data and the connection with the real market. The more sensitive the company will be to capture possible business opportunities, a powerful guarantee for corporate economic growth. However, an excessive analysis may break away from the actual business’s needs and increase the enterprise’s decision-making cost. At the same time, excessive superstition on data can easily lead to the misunderstanding of “excessive specialisation” and reduce the service value of BDC to the enterprise.

The last, big data insight and prediction capability enable companies to realise real-time insights into the environment and market demand, foresee changes in the environment, and then adjust production plans and service processes promptly, which is beneficial to expand the market and increase revenue growth. Raguseo and Vitari (2018) also point out that big data analysis help enterprises create greater business value, thereby driving the growth of company performance. However, relying too much on big data insight and prediction capability will neglect the impact of other factors such as policy environment on the development of enterprises, which is ultimately detrimental to the improvement of enterprise performance.

Combined with the above analysis, BDC help enterprises integrate big data resources and effectively deploy technology and talent for data analysis to realise internal process optimisation and external competitive trend prediction, forming valuable insights and generating insights. However, excessive BDC often leads to higher investment, resulting in the “IT capability paradox” trap, leading to the decline of enterprise financial performance and deviation from the organisation’s original goal. Therefore, BDC and enterprise financial performance may present the nonlinear “Converse-U” relationship. Based on the construct dimension of BDC, it can comprehensively and accurately reflect the financial performance as a whole, therefore this paper puts forward the following research assumption:

**H1.** BDC has a “Converse-U” effect on financial performance

### Big Data Capability and Strategic Flexibility

Strategic flexibility is a dynamic capability transformation process in the view of the organisational concept. It is a consistent capability that matches the degree of environmental changes based on the original strategy (Li et al., 2010; Nadkarni and Herrmann, 2010), realising the response and dynamic adjustment of the organisation to environmental changes, and achieving the high performance of the whole organisation (Weihong et al., 2013; Farnese et al., 2016). According to characteristics of strategic flexibility, its dimension is divided into preemptive flexibility and response flexibility (Hai and Dong, 2010). On the other hand, according to dynamic capability theory, strategic flexibility dimension is divided into capability flexibility and resource flexibility (Matthyssens et al., 2005; Tennan et al., 2010). Lastly, according to resource utilisation and process allocation, it can be divided into resource flexibility and coordination flexibility (Zhengang et al., 2021). Researches have been studied on the relationship between BDC and strategic flexibility from data empowerment (Ziqing, 2021) and the digital economy (Katsuhiko and Michael, 2004). According to Tennan et al. (2010), this paper studies the mechanism of BDC on strategic flexibility from two aspects of resource flexibility and capability flexibility.

According to the dynamic capability view, in the dynamic changing market, technology and institutional environment, the limited nature of the enterprise’s resources can hardly meet the changing demand of the market, and the strategic flexibility can quickly allocate its resources or seek new resources to benefit the enterprise (Sanchez, 1997). As a dynamic capability, BDC props enterprises to integrate internal and external resources, deeply analyse the integrated environment, and formulate specific strategies (Weihong et al., 2016). Strategic flexibility, as a potential capability including capability flexibility and resource flexibility, enables firms to adapt quickly to dynamic environments when faced with rapid changes in internal and external environments (Matthyssens et al., 2005; Tennan et al., 2010). Capability flexibility refers to the ability to discover new resources and allocate them reasonably in the face of environmental uncertainty to maximise the utilisation of resources (Cingöz and Akdoðan, 2013). Compared with other competitors, enterprises with high flexibility can better identify and grasp opportunities in a rapidly changing environment, complete the updating and transformation of relevant resources, timely adjust enterprise strategies, and then create advantages to boost economic benefits. Resource flexibility is low-cost and rapid scheduling of resources to meet consumers and the market (Beard, 1984). Enterprises with high resource flexibility can actualise a high sharing of resources, which can also create value for enterprises (Han and Zhang, 2021). Resource flexibility enables the enterprise to transform from one resource to another at low cost, enriching the resource combination and constructing a unique competitive advantage, thereby improving enterprise financial performance.

Based on the above analysis, BDC is conducive to the adaptability of organisations in an uncertain environment, promoting the improvement of capability flexibility and resource flexibility, and further enhancing financial performance. However, excessive BDC, such as paying too much emphasis on big data resource construction and deployment of big data analysis technology, may lead to the failure of the effective integration of big data capabilities with the original business, affecting the construction of strategic flexibility. Therefore, the following research assumption is proposed:

**H2.** BDC has a “Converse-U” effect on strategic flexibility.

### Strategic Flexibility’s Mediating Effect

The direction and extent of impact between BDC and financial performance can be measured directly, but the relationship can be a complex feedback system. As an important dynamic capability of an organisation, BDC adapts to the dynamic environment based on organisational perspective and forms strategic flexibility. Strategic flexibility is considered in the research process, which itself is also a process of strategic flexibility formation and will affect enterprise financial performance. In this study, BDC affects strategic flexibility and, thus, financial performance. Therefore, the following research assumption is proposed:

**H3.** Strategic flexibility plays an intermediary role in the “Converse-U” relationship between BDC and financial performance.

### Environmental Dynamics’ Moderating Effect

Environmental dynamics are the speed and unpredictability of environmental change (Lazonick and Prencipe, 2008). Based on the market and technology perspective, studies have been carried out on technological and external market environment dynamics (Su, 2011; Sainio et al., 2012). In contrast, less attention has been paid to the influence of institutional environment dynamics. The institutional environment significantly impacts development strategy, speed, and scale (Yingwei et al., 2019). A loose and favourable institutional environment is more conducive to BDC development and the consolidation of competitive advantages (Zhang et al., 2020). Therefore, in this paper, environmental dynamics includes three dimensions: technological environment dynamics, market environment dynamics, and institutional environment dynamics. In the context of the dynamic environment, strategic flexibility is a key factor affecting organisational performance. Strategic flexibility improves internal communication efficiency and strategic execution ability, thus positively affecting financial performance. Wamba et al. (2017) confirm that environmental dynamics regulate the relationship between organisational BDC and supply chain agility. Based on a double marginalised perspective, environmental dynamics can positively regulate the relationship between environmental and financial performance (Kim et al., 2011). In a highly dynamic environment, enterprises rely more on the advantages of deep analysis, trend insight, and demand prediction brought by big data capabilities to maintain corporate performance at a higher level. Based on the above analysis, the following assumption is proposed:

**H4.** Environment dynamics plays a positive role in the “Converse-U” relationship between BDC and strategic flexibility. Under high environmental dynamics, the “Converse-U” impact of BDC on strategic flexibility is weaker.

**FIGURE 1:**
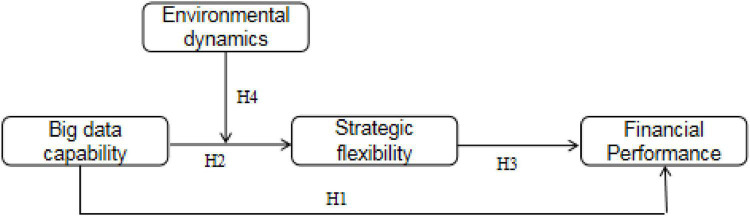
Research model.

According to the previous literature, this paper constructs a hypothetical model of the effect mechanism of BDC on financial performance, as shown in Figure 1.

## Research Methods

### Research Sample and Process

Our research uses questionnaires in 10 provinces: Shanghai, Beijing, Jiangsu, Anhui, Zhejiang, Shanxi, Liaoning, Gansu, Chongqing, and Guizhou. The industries were distributed in the fields of telecommunication, energy, high-tech, and consulting services. We avoided possible common method variance, and the samples were paired to collect data from different sources. The head of the enterprise big data department (if there was no big data department, the informatisation department could be responsible for it) reported the BDC, strategic flexibility, and environmental dynamics. The financial executive reported the financial performance. The big data manager and the financial executive formed a set of research questionnaires after pairing. The questionnaire included an electronic questionnaire and an on-site questionnaire. For the questionnaires filled on-site, the identity of the interviewees would be confirmed by the General Manager’s Office. Then, the questionnaire data of big data managers and financial executives needed to be collected simultaneously to directly match into a set of questionnaires. Electronic questionnaires were mainly aimed at the situation that managers cannot be present simultaneously. The General Manager Office was requested to provide names and email addresses of the two managers, and the investigators sent the questionnaire to the manager, who was required to fill in the questionnaire and return it. Throughout the survey, it was repeatedly emphasised that the survey is voluntary to avoid unnecessary deviations.

In this study, 400 big data department manager questionnaires and 400 financial department executive questionnaires were distributed. Three hundred two questionnaires were collected from big data department managers, with a recovery rate of 75.5%; 310 financial department executives’ questionnaires were collected, with a recovery rate of 77.5%. After eliminating the low-quality questionnaires that did not meet the research requirements, 274 questionnaires that finally met the research design were collected, with an effective rate of 68.5%. Based on the analysis of valid samples, the statistical data are as follows: (1) The nature of organisation: the largest number of private enterprises accounts for 51.1%, local state-owned enterprises account for 26.3%, Hong Kong, Macao and Taiwan independence assets and holding enterprises account for similar proportion with foreign-funded enterprises. The overall distribution is relatively balanced, with a certain extent of universality. (2) Organisation type: information transmission, software and technology services industries account for the highest proportion of 35.8, the manufacturing industry is 29.9%, and other industries are not much different. (3) Organisation scale: this research takes the number of employees as the index to measure the organisation scale, enterprises with 301–1,000 employees account for 35.8%, enterprises with 300 employees and below account for 24.8%, and enterprises with 1,001–2,000 account for 17.5%. The overall distribution is balanced and has certain representativeness. (4) Annual sales: 33.2% of enterprises with sales of more than 300 million, 26.3% of enterprises with sales between 30 million and 100 million, and the others with similar sales. The specific sample composition is shown in Table 1.

**TABLE 1:** Descriptive statistics of the sample and respondents.

Factor	Classification	Sample (*N* = 274)	Proportion (%)
Industry	Mining, construction	13	4.7
	Electricity, gas and water production and supply industries	6	2.2
	Transportation, warehousing and postal services	34	12.4
	Financial industry	15	5.5
	Agriculture, forestry, animal husbandry and fishery	4	1.5
	Wholesale and retail trade	12	4.4
	Information transmission, software and technology services	98	35.8
	Manufacturing industry	82	29.9
	Accommodation and catering	4	1.5
	Other	6	2.2
Firm size (number of employees)	<300	68	24.8
	301–1,000	98	35.8
	1,001–2,000	48	17.5
	2,001–3,000	14	5.1
Annual sales	<30 million	41	15.0
	30–100 million	72	26.3
	100–200 million	42	15.3
	200–300 million	28	10.2
	>300 million	91	33.2
Ownership	Central enterprises (including state-owned holding)	27	9.9
	Local state-owned enterprises (including state-owned holding)	72	26.3
	Private enterprises (including private holding)	140	51.1
	Hong Kong, Macao and Taiwan independence assets and holding enterprises	16	5.8
	Wholly foreign-owned and controlled enterprises	17	6.2
	Other	2	0.7

*Industrial division refers to National Economy Industry Classification.*

### Variable Measurement

To ensure the effective measurement of the research model, firstly, the scale selected in this study comes from the mature scale of the top journals at home and abroad. Graduate students with excellent English proficiency are invited to translate the English scale to ensure quality. For items in doubt, experts in the same field are invited to confirm again, and the initial version of the questionnaire is formed. Secondly, other scholars in the same field are invited to back-translate the original questionnaire. The back-translated English questionnaire is compared with the original questionnaire to further modify the existing problems. Thirdly, five executives are invited to conduct a pre-search to discuss and revise the unclear and ambiguous questions. Finally, the questionnaire for conducting a large sample survey is confirmed. The Likert Scale measures variables (1 = totally disagree; 2 = disagree; 3 = uncertain; 4 = agree; 5 = totally agree).

(1) Big Data Capability (BDC). This paper draws on the situation scale developed by Kim et al. (2011) and Weihong et al. (2016), including three dimensions: big data acquisition integration capability, big data in-depth analysis capability, and big data insight and prediction capability, with a total of 12 items. Among them, big data acquisition and integration capability dimension include four items, mainly referring to the scale of Kim et al. (2011), to measure the enterprise’s capability of big data resources acquisition and data processing, such as “The enterprise has infrastructure related to big data, including big data support platform and business application system.” Big data in-depth analysis capability dimension includes four items, mainly referring to the research of Weihong et al. (2016) and Lin and Kunnathur (2019), to measure the enterprise’s ability to excavate latent value from the magnanimous data through analysis. Representative items such as “Enterprises can effectively clean and standardise massive data.” Lastly, big data insight and prediction capability dimension include four items, mainly referring to the research of Gang and Min (2014) and Lin and Kunnathur (2019), to measure the real-time understanding and trend prediction capability of market and environment based on big data. Representative items include “Enterprises realise real-time insight and trend prediction on the market based on big data.” In this study, the Cronbach’s Alpha for the BDC scale is 0.949.
(2) Strategic flexibility. Strategic flexibility includes two dimensions: resource flexibility and capability flexibility. This paper draws on the strategic flexibility scale of Grewal and Tansuhaj (2001) and Li et al. (2010), with a total of six items, which has been widely used in the research of Chinese situation (Tennan et al., 2010). Among them, three items are used to measure the dimension of resources flexibility, and the representative items include “The difficulty and cost of enterprise resources transforming from one use to another are low.” On the other hand, three items measure the dimension of capability flexibility, and the representative items include “The enterprise can identify the change of external environment and transform the use of resources.” The Cronbach’s Alpha for the strategic flexibility scale is 0.904.
(3) Environmental dynamics. Three dimensions consist of environmental dynamics, including market, technical, and institutional environmental dynamics. This paper draws on the scale of Jansen et al. (2006) and Jintong-Tang et al. (2010), with a total of six items. The scale has already been applied in the research of the Chinese context (Chunyan and Shuming, 2010; Wencong and Guilong, 2011; Jinfeng et al., 2019), which ensures the effectiveness of the measurement. Among them, two items measure the dynamic dimension of the market environment. The representative item includes “In the enterprise’s business scope, the customer’s product/service preference changes rapidly.” Two items measure technical, environmental dynamics, for instance, “the technical innovation speed is fast in industry field.” Two items measure the institutional environmental dynamics, the representative item includes “Governments at all levels have various assistance and policies for the enterprise’s BDC building.” In this study, the Cronbach’s Alpha of the environmental dynamics scale was 0.856.
(4) Financial performance. Financial performance includes four dimensions: profitability, sales growth rate, customer retention, and return on investment. This paper draws on Li et al. (2010) and Akter et al. (2016). There are four items in total: “Adoption of big data capability improves customer retention” and “Adoption of big data capability improves the sales growth rate.” The financial performance scale in this study has a Cronbach’s alpha of 0.849.
(5) Control variables. Based on previous studies, this study selected industry type, firm size, annual sales, and ownership as control variables (Akter et al., 2016; Weihong et al., 2016). This paper sets four control variables as virtual variables, assigning different values to different types and ranges of options.

### Validity and Reliability Analysis

The factor loading, average variance extraction (AVE), and composite reliability (CR) of each variable are calculated to test the scale’s validity. Results show that factor loading of each item in the scale is between 0.6 and 0.9, indicating that potential variables have a strong explanatory ability to measure variables. Moreover, AVE of BDC, strategic flexibility, environmental dynamics, and financial performance is 0.613, 0.589, 0.613, and 0.501, respectively, which is higher than the critical value of 0.5. The CR of each variable is 0.950, 0.851, 0.905, and 0.857, respectively, which is higher than the critical value of 0.8, indicating that the measurement of the four variables has satisfactory convergent validity. The intrinsic quality of the model is acceptable, as shown in Table 2.

**TABLE 2:** Assessment of reliability and validity of reflective constructs.

Variable	Measures	Factor loading	AVE	CR
Big data capability (BDC)	BDC1: Enterprise has infrastructure related to big data, including big data support platform and business application system, etc.	0.692	0.613	0.950
	BDC2: Enterprises can continuously obtain and update internal and external big data information in time	0.702		
	BDC3: Enterprises can quickly store and process large amounts of data	0.769		
	BDC4: Enterprises can continuously learn and update big data technology (data crawling, storage, analysis, visualisation)	0.755		
	BDC5: Enterprises can effectively clean and standardise massive data	0.748		
	BDC6: Enterprises are skilled in using technologies, tools and platforms related to distributed computing of big data (Hadoop/HPCC/Storm/Pentaho BI etc.)	0.826		
	BDC 7: Enterprises can analyse unstructured data such as text and voice in real-time	0.810		
	BDC 8: Enterprises can mine valuable information from massive customer and market information	0.779		
	BDC 9: Enterprises know and predict customer behaviour in real-time based on big data	0.823		
	BDC10: Enterprises predict the behaviour of partners and competitors based on big data	0.791		
	BDC11: Enterprises realise real-time insight and trend prediction on the market based on big data	0.812		
	BDC12: Enterprises identify business operation and development strategies based on big data	0.870		
Financial performance (FP)	FP1: Adoption of big data capability improves profitability	0.716	0.589	0.851
	FP2: Adoption of big data capability improves sales growth rate	0.724		
	FP3: Adoption of big data capability improves customer retention	0.759		
	FP4: Adoption of big data capability improves the sales growth rate	0.862		
Strategic flexibility (SF)	SF1: Difficulty and cost of enterprise resources transforming from one use to another is low	0.796	0.613	0.905
	SF2: Time of enterprise resources transforming from one use to another is less	0.762		
	SF3: Effective usable range of enterprise existing resources is relatively wide	0.753		
	SF4: Enterprise can identify the change of external environment and transform the purpose of resources	0.788		
	SF5: Enterprises are always able to allocate resources appropriately to cope with changing environments	0.775		
	SF6: Enterprise can effectively allocate resources through organisational systems and procedures according to objectives	0.823		
Environmental dynamics (ED)	ED1: In the enterprise’s business scope, the customer’s product/service preference changes rapidly	0.621	0.501	0.857
	ED2: Many new customers are emerging to buy enterprise products/services	0.771		
	ED3: The technical Innovation speed is fast in the industry field	0.716		
	ED4: Technological change offers great opportunities for the whole industry	0.701		
	ED5: Governments at all levels have various assistance and policies for the enterprise’s BDC building	0.697		
	ED6: The enterprise has access to a variety of information about big data	0.733		
X^2^/df = 2.343, RMSEA = 0.070, IFI = 0.912, TLI = 0.902, CFI = 0.911				

To ensure the reliability and validity of the study, AMOS 23.0 statistical software is utilised to conduct confirmatory factor analysis on four variables. Six-factor combination model data are obtained by establishing different factor models composed of BDC, strategic flexibility, financial performance and environmental dynamics, and the analysis results are shown in Table 3. Compared with other models, the four-factor model has the best fitting effect on data. Specifically, from single-factor model to four-factor model, X^2^(df) decreases from 1713.823 to 805.976, X^2^/df decreases from 4.897 to 2.343, root-mean-square error of approximation (RMSEA) decreases from 0.119 to 0.070, standardized root mean square residual (SRMR) decreases from 0.094 to 0.049. The smaller values of these indexes are, the better fitting effect is. In addition, comparative fit index (CFI) increases from 0.737 to 0.911, incremental fit index (IFI) increases from 0.739 to 0.912, and tucker-lewis index (TLI) increases from 0.716 to 0.902. The closer these three indicators are to 1, the better the model is. On the whole, the fitting indexes of the four factors model are better than other models, indicating that BDC, strategic flexibility, financial performance and environmental dynamics are disparate concepts.

**TABLE 3:** Analysis of confirmatory factors.

Model	X^2^ (df)	X^2^/df	CFI	IFI	TLI	RMSEA	SRMR
Single factor model (BDC+SF+FP+ED)	1713.823	4.897	0.737	0.739	0.716	0.119	0.094
Two-factor model (BDC+SF+FP, ED)	1296.689	3.715	0.817	0.818	0.802	0.100	0.071
Three-factor model 1 (BDC, SF+FP, ED)	1096.451	3.151	0.856	0.857	0.843	0..089	0.066
Three-factor model 2 (BDC+SF, FP, ED)	1013.828	2.913	0.872	0.872	0.861	0.084	0.057
Three-factor model 3 (BDC+FP, SF, ED)	1115.427	3.205	0.852	0.853	0.839	0.090	0.068
Four-factor model (BDC,FP, SF, ED)	805.976	2.343	0.911	0.912	0.902	0.070	0.049

## Data Analysis and Hypothesis Testing

### Correlation Analysis

In this paper, SPSS23.0 statistical software is used for data analysis, and the correlation analysis results of each variable are shown in Table 4. BDC, strategic flexibility, environmental dynamics, and financial performance are significantly positively correlated, which provides preliminary support for the follow-up study on the relationship between variables.

**TABLE 4:** Correlation matrix of variables.

Variable	X1	X2	X3	X4	X5	X6	X7	X8
Industry	1							
Firm size	−0.147*	1						
Annual sales	–0.116	0.685**	1					
Ownership	−0.160**	−0.166**	−0.136*	1				
Big data capability	0.156**	0.221**	0.330**	−0.135*	1			
Strategic flexibility	0.146*	0.125*	0.241**	–0.096	0.784**	1		
Environmental dynamics	0.095	0.135*	0.172**	–0.035	0.473**	0.409**	1	
Financial performance	0.130*	0.136*	0.094	–0.007	0.528**	0.510**	0.383**	1
Mean value	3.672	2.533	3.204	2.745	4.1128	3.9775	4.1381	4.1451
Standard deviation	2.438	1.364	1.503	0.984	0.649	0.589	0.520	0.577

Significance level: ***means 0.1%, **means 1%, and *means 5%.

### Hypothesis Testing

(1) Main effect testing: Hypothesis1 puts forward that the BDC has a “Converse-U” effect on financial performance. Financial performance is set as the dependent variable in the research. Four control variables, such as industry type, firm size, annual sales, and ownership, are added to the model. Then BDC’s first-power term and quadratic term are added to test the hypothesis. To reduce the possibility of multiple co-linear relationships caused by nonlinear paths in the model, the quadratic term of BDC is decentralised and analysed according to the curve effect test procedure suggested by Porter et al. (1994) and Hayes and Preacher (2010). The results of hierarchical regression analysis are shown in Table 5. Model 2 and model 3 show that quadratic term of BDC hurts financial performance (β = -0.173, *p* < 0.05). In addition, the first-power term of BDC has a significant positive impact (β = 0.407, *p* < 0.001), indicating that BDC are positively associated with financial performance, and the relationship between two is “Converse-U,” Hypothsis1 is supported.

**TABLE 5:** Regression test results of main effect and the mediating effect.

Variable		Financial performance					Strategic flexibility		
		Model 1	Model 2	Model 3	Model 4	Model 5	Model 6	Model 7	Model 8
Control variable	Industry type	0.038*	0.014	0.009	0.008	0.018	0.040*	0.004	0.001
	Firm size	0.069**	0.068*	0.070*	0.076*	0.082**	–0.026	–0.028	–0.026
	Annual sales	0.003*	−0.070*	−0.078**	−0.079**	−0.056*	0.116	0.011	0.006
	Ownership	0.028	0.047	0.035	0.036	0.040	−0.024***	0.004	–0.004
Independent variable	BDC		0.492***	0.407***	0.255**			0.714***	0.659***
	BDC^2^			−0.173*	−0.148*				−0.112*
Mediation variable	Strategic flexibility				0.231**	0.505***			
Regression index	*R* ^2^	0.044	0.305	0.320	0.341		0.092	0.618	0.624
	Adj-R^2^	0.030	0.292	0.305	0.324		0.078	0.611	0.616
	*F*-value	3.083	23.482	20.960	19.668		6.785	86.704	73.916

Significance level: ***means 0.1%, **means 1%, and *means 5%.

(2) Mediating effect testing: It is assumed that strategic flexibility mediates the relationship between BDC and financial performance. According to the moderated mediation effect test method proposed by Zhonglin et al. (2004), three conditions should be met: (1) BDC is significantly correlated with financial performance, (2) BDC is significantly related to strategic flexibility, and (3) when strategic flexibility enters into the relationship between BDC and financial performance, the relationship between BDC and financial performance is not significant, and strategic flexibility and financial performance are significant, which is a complete intermediary role. If the relationship between the two is still significant but weakened, then strategic flexibility plays a part in mediating roles. In step one, based on control variables, Model 3 tests the main effect of BDC on financial performance, and the result is verified, and Step two continues to test the influence of BDC on strategic flexibility, the intermediary variables. Model 7 and Model 8 show that quadratic term of BDC is significantly negatively correlated with the strategic flexibility (β = -0.112, *p* < 0.05), and the first power of BDC is positively correlated with the strategic flexibility (β = 0.659, *p* < 0.001), hypothesis 2 is verified. In Step three, strategic flexibility is put into the main effect relationship and Model 4 shows that the regression coefficient of BDC quadratic term on financial performance is significant (β = -0.148, *p* < 0.05), and the financial performance in model 6 is also significantly positively affected by strategic flexibility (β = 0.505, *p* < 0.001), indicating that strategic flexibility has the partial mediating effect, assuming data support 3. To further demonstrate the mediating effect of strategic flexibility, the bootstrap method performed repeat sampling to expand the sample size to 5,000 with a 95% confidence interval. The specific result is shown in Table 6. The coefficient of strategic flexibility is 0.23, and the confidence interval did not include 0 (0.074, 0.387). Therefore, the mediating role of strategic flexibility between BDC and financial performance is established, and H3 is verified.

**TABLE 6:** Bootstrap analysis of mediating effects.

Path	Effect value	Standard error	95% CI	
			Lower limit	Upper limit
BDC-SF-FP	0.230	0.079	0.074	0.387

(3) Moderating effect testing: Establish a regulatory mediation model and test the regulatory effect of environmental dynamics according to the method recommended by Edwards and Lambert (2007). After controlling industry type, firm size, annual sales, and ownership, the interaction items of BDC and environmental dynamics are decentralised and then brought into the model to construct the first-power item and quadratic interaction item of BDC, as shown in Table 7, in Model 4, the quadratic interaction item of BDC and environmental dynamics is significantly positive (β = 0.228, *p* < 0.05), indicating that strategic flexibility plays a regulatory role in the “Converse-U” relationship between BDC and financial performance.

**TABLE 7:** Moderating effect analysis.

Variable		SF			
		Model 1	Model 2	Model 3	Model 4
Control variable	Industry type	0.040***	0.009	0.005	0.002
	Firm size	–0.026	–0.018	–0.025	–0.029
	Annual sales	0.116***	0.048	0.042	0.038
	Ownership	–0.024	–0.051	–0.049	–0.048
Argument	BDC^2^		−0.560***	−0.486***	−0.399***
Adjusting variable	ED			0.292***	0.169*
Interactions	BDC^2^ × ED				0.228*
Regression index	R^2^	0.092	0.325	0.384	0.395
	Adj-*R*^2^	0.078	0.312	0.370	0.379
	*F*-value	6.785	25.753	27.751	24.834

Significance level: ***means 0.1%, **means 1%, and *means 5%.

**FIGURE 2:**
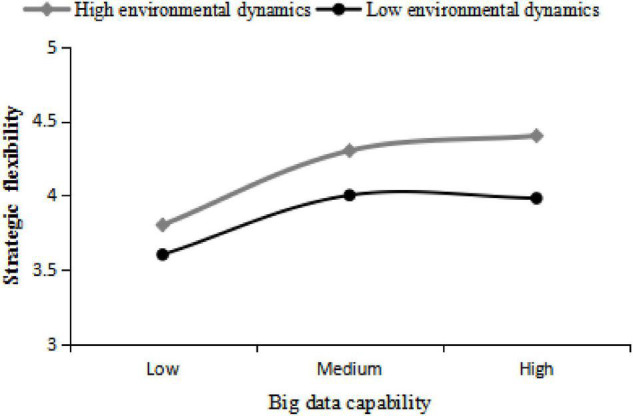
Moderating effect of environmental dynamic.

Simple slope analysis is continued to further explain the moderating effect of environmental dynamics (Aiken and West, 1991). The part whose environmental dynamics are less than its mean minus standard deviation is divided into the low correlation group (0–3.619). The mean plus standard deviation part is divided into a high correlation group (4.658–5). Results show that environmental dynamic positively moderates the “Converse-U” relationship between BDC and financial performance. As shown in Figure 2, the higher environmental dynamics is the weaker “Converse-U” impact of BDC on financial performance. In other words, the negative impact of excessive BDC on financial performance is reduced, and the critical point is at a higher level of BDC. It can be seen that the high-dynamic environment is more conducive for enterprises to exert the positive effect of BDC on financial performance to a greater extent, which verifies hypothesis 4.

## Conclusion

Based on dynamic capability theory, this paper verifies the relationship between BDC and financial performance in the context of digital transformation in COVID-19 and the mediating effect of strategic flexibility and the regulating mechanism of environmental dynamics. Results show that BDC has a “Converse-U” effect on financial performance, further expanding the existing research conclusions on the relationship between BDC and financial performance. Previous studies have confirmed a positive linear relationship between BDC and financial performance (Barton and Court, 2012; Chen et al., 2015). This paper verifies the nonlinear relationship between BDC and financial performance based on a previous study. Further, it explains the key factors that drive the improvement of financial performance and the contextual factors that BDC plays a significant role. The results show that appropriate utilisation of data platforms and emerging technology would be conducive to completing the collection and integration of tangible and intangible resources from inside and outside, mining data value and getting insight into the relationship between the real market to better grasp the customer demand and potential business opportunities, and finally function in profitability increase, customer maintenance level improvement, and sales rate growth. That means strengthening the ability to integrate and utilise big data resources to improve performance has become a crucial part of business management in the environment of data explosion. Secondly, BDC has a “Converse-U” effect on strategic flexibility. Strategic flexibility is an important transmission mechanism of the impact of BDC on financial performance and plays an intermediation role in the “Converse-U” relationship between BDC and financial performance. Previous studies tend to focus on the direct effect of BDC on financial performance, ignoring the possible mechanism between BDC and financial performance. This paper verifies the role of strategic flexibility, which provides a theoretical basis for the positive effect of BDC on financial performance through the development of strategic flexibility in a dynamic environment. Therefore, enterprises should be equipped with flexible resources and capabilities to meet the needs of business activities and possess the capability to efficiently utilise their resources and seek new resources to consolidate and strengthen competitive advantages. Thirdly, environmental dynamics positively regulates the relationship between BDC and financial performance, causing the “Converse-U” relationship to be flat, weakening the negative impact of excessive construction of BDC on financial performance. Higher environmental dynamics mean increasing unpredictability, while big data technology in-depth analysis of massive data makes enterprises better cope with environmental challenges. Big data insight and prediction capability complete market trend tracking and make it possible for enterprises to launch products or services that satisfy market demands before competitors, thus consolidating market position and improving competitive advantages. Therefore, a high dynamic environment is more conducive to playing a positive role of BDC on financial performance.

The relevant conclusions of this study can provide some meaningful implications for enterprises in the COVID-19 era: First, in the context of COVID-19, big data and technology have demonstrated strong advantages in improving enterprise management and services. On the one hand, the rapid development of the online economy caused by COVID-19 has greatly enriched the data sources of enterprises, facilitated their access to comprehensive and accurate industry data, and optimised them to cope with risks and challenges. On the other hand, BDC enables enterprises to rapidly update products and services based on market changes, personalised marketing, consummate pricing systems, optimise manufacturing and service processes, strengthen dynamic capability, and eventually elevate environmental adaptability and adjustment capability of enterprises. Enterprises can encourage activities related to big data, such as data mining, integrated statistics, model prediction, and other activities to analyse internal and external data to find potential market opportunities. It is essential to construct BDC appropriately, such as digital transformation in all aspects of production and service. Amount of time and money will be invested in constructing an enterprise’s excessive BDC, which will lead to less investment and attention on other aspects.

Meanwhile, the matching and coordination between BDC and capabilities required by the original business cannot be considered, resulting in a decline in the enterprise’s financial performance. Over-emphasis on BDC building is more likely to fall into the paradox of “capability trap.” Therefore, in the construction and management of BDC, strengthening the matching degree with the original business and capabilities and then carrying out appropriate management and control to enhance financial performance is essential.

Next, enterprises should pay attention to the crucial role of strategic flexibility in the integrative development of big data. The sudden outbreak of COVID-19 has brought about rapid changes in the market environment, with many enterprises facing the dilemma of increased operational risks and resource constraints, making it difficult for the enterprise to complete operating activities based on the existing resources inhibiting the financial performance advance. Enterprise managers should construct an effective strategic flexibility formation mechanism and, moreover, promote organisational flexibility in human resources, corporate culture, and organisation structure by cultivating emotional core competence. Attention is needed to be paid to excavating the potential value of existing capacity and resources, rationally allocating existing resources, and absorbing new resources to strengthen strategic flexibility. In resource flexibility, on the one hand, the enterprise should weaken the degree of dependence on resources and actively look for alternative resources, such as diversification of upstream and downstream suppliers, talent introduction, and training.

On the other hand, enterprises need to increase the degree of resources transformation, cater to the current market demand, establish market development and product research team to clarify market dynamics, and demand status in real-time. In terms of capability flexibility, dynamic capability should be established to improve the comprehensive capability of enterprises in response to emergencies. In the context of COVID-19, factors affecting flexibility, such as good cash flow and financial status, can help enterprises improve their coping ability and weather rapid changes in the external environment.

In the context of big data, companies should rationally view environmental dynamics, fully realise that environmental fluctuations are both opportunities and challenges, and should adopt a variety of flexible ways to avoid the adverse effects of institutional support and market strategy lag on organisational operations and then promote the organisation performance improvement—the suddenness of COVID-19 tests whether enterprises can form resilience in adverse events. Firms can take the opportunity to upgrade their business models and promote the corporate transformation to form key competitive advantages. The practice has shown that environmental dynamics negatively correlate with corporate performance (Fuli and Shuhong, 2019), especially in high dynamic conditions. The unpredictability of the financial markets and environment urges enterprises to use the results of data analysis to gain insight into development trends, constantly adjust strategic goals and plans, and ensure the adaptability of the BDC cultivation process to environmental changes. In the context of COVID-19, the decline and delay in demand hit the survival and development of enterprises, especially small and medium-sized enterprises. As a result, accelerating digital transformation and propelling business model upgrade is the key to promoting business recovery. Big data technology should be utilised to realise accurate analysis and deep exploration of market trends and business opportunities.

Finally, it is important to note that the research sample is not limited to specific industries. Comparative research can be carried out for some specific industries to increase the persuasiveness of the research conclusions in the future. Secondly, this research is based on cross-sectional data. Without carrying out longitudinal dynamic evolution analysis on the relationship between variables, further research can be conducted to reveal the dynamic relationship among BDC, strategic flexibility, and financial performance through long-term tracking investigation. Thirdly, further enrich contextual variables of BDC’s research, such as corporate culture and organisational learning, and further explore other mediating variables and regulating mechanisms. It is also possible to compare the impact of BDC on financial performance in China and other countries. These are the research directions that can be further explored in the future.

## Data Availability Statement

The raw data supporting the conclusions of this article will be made available by the authors, without undue reservation.

## Ethics Statement

The studies involving human participants were reviewed and approved by the Nanjing University of Posts and Telecommunications. The patients/participants provided their written informed consent to participate in this study.

## Author Contributions

LC: data curation and empirical analyses. LZ: writing the manuscript and mentoring. WZ: writing the manuscript and methodology. GL: writing the manuscript and visualisation. JZ: reviewing the manuscript and conceptualisation. YZ: reviewing the manuscript and validation. All authors contributed to the article and approved the submitted version.

## Conflict of Interest

The authors declare that the research was conducted in the absence of any commercial or financial relationships that could be construed as a potential conflict of interest.

## Publisher’s Note

All claims expressed in this article are solely those of the authors and do not necessarily represent those of their affiliated organizations, or those of the publisher, the editors and the reviewers. Any product that may be evaluated in this article, or claim that may be made by its manufacturer, is not guaranteed or endorsed by the publisher.
