# Corrigendum: Financial Performance Under the Influence of the Coronavirus Disease 2019: Effects of Strategic Flexibility and Environmental Dynamics in Big Data Capability

**DOI:** 10.3389/fpsyg.2022.914904

**Published:** 2022-05-10

**Authors:** Limei Chen, Liping Zhai, Weiwei Zhu, Gongzhi Luo, Jing Zhang, Yaozhen Zhang

**Affiliations:** School of Management, Nanjing University of Posts and Telecommunications, Nanjing, China

**Keywords:** COVID-19 pandemic, big data capability, strategic flexibility, financial performance, environmental psychological dynamics

In the original article the authors neglected to include a Funding statement. The correct Funding statement is included below.

## Funding

This study received funding from Postgraduate Research & Practice Innovation Program of Jiangsu Province (KYCX20_0850) to LZ.

The authors apologize for this error and state that this does not change the scientific conclusions of the article in any way. The original article has been updated.

## Publisher's Note

All claims expressed in this article are solely those of the authors and do not necessarily represent those of their affiliated organizations, or those of the publisher, the editors and the reviewers. Any product that may be evaluated in this article, or claim that may be made by its manufacturer, is not guaranteed or endorsed by the publisher.

